# The effect of high-frequency rTMS over left DLPFC and fluid abilities on goal neglect

**DOI:** 10.1007/s00429-024-02770-y

**Published:** 2024-03-22

**Authors:** Gizem Arabacı, Batuhan S. Cakir, Benjamin A. Parris

**Affiliations:** Department of Psychology, Bournemouth University, Talbot Campus, Fern Barrow, Poole, Dorset UK

**Keywords:** High frequency, rTMS, Goal neglect, DLPFC, Working memory

## Abstract

Goal neglect refers to when an aspect of task instructions is not utilised due to increased competition between goal representations, an attentional limit theoretically linked to working memory. In an attempt to alleviate goal neglect and to investigate the association between dorsolateral prefrontal cortex (DLPFC)-supported working memory and goal neglect, we used high-frequency repetitive transcranial magnetic stimulation to the left DLPFC whilst participants completed the letter-monitoring task, a measure of goal neglect, and an N3-back task, a working memory task known to be affected by rTMS of the left DLPFC, following 20 min of active and sham stimulation (run on separate days). We found increased accuracy on the N3-back task in addition to decreased goal neglect in the active compared to sham condition when controlling for age and fluid abilities (as assessed by matrix reasoning performance). Furthermore, analysis showed that active stimulation improvements on both the N3-back and letter-monitoring tasks were greater for those with higher fluid abilities. These findings provide support for the link between the DLPFC-support working memory and goal neglect. Increased performance on the N3-back task also supports the literature reporting a link between left DLPFC and verbal working memory. Results are evaluated in the context of potential use to alleviate symptoms of disorders related to goal neglect.

## Introduction

Working memory is one of the three core components of executive functions along with inhibition and switching (Friedman and Miyake 2017). Working memory is responsible for activating and maintaining information that is not available in the environment for as long as the information is needed (Baddeley and Hitch 1994). Some definitions also include resisting interference from irrelevant information to enable the maintenance of relevant information (Conway and Engle 1994; Kane and Engle 2000, 2002).

Duncan et al. (2008) introduced what they referred to as a different form of attentional capacity that they theoretically linked to working memory, specifically the episodic buffer of working memory system. Duncan et al. suggested that the instructions of a task need to be translated into goals and that all goal-relevant information has to be accessible throughout the task in what they called the *task model*. Due to the limited capacity of the task model, as the complexity of the instructions increases, some of the information is lost or inappropriately weighted, leading to *goal neglect*, especially in those with lower fluid intelligence.

In goal neglect, despite being able to report the instructions before and after the task, participants neglect some of the instructions during task performance (Duncan et al. 2008). Using a letter-monitoring task, the occurrence of goal neglect has been reported in older adults with lower fluid intelligence and in frontal lobe patients with impaired fluid intelligence (Duncan et al. 2008) but also in younger adults with inattention, even when fluid intelligence was controlled (Arabaci and Parris 2019; Elisa et al. 2016).

In the letter-monitoring task, a pair of letters or digits are presented either side of a centrally present dot on a computer screen. Following an initial written cue, participants are instructed to read out either the left or right letter from pairs of letters and ignore digits for a number of successively presented pairs, before they are presented with another cue. The cue may require switching to the unattended side or stay focussed on the same side. Goal neglect is measured as the failure to follow the second cue-instructed goal. When the cue indicates attending to the side being currently attended, it is referred to as a repeat trial. When the cue requires changing the attended side, it is referred to as a switch trial. Therefore, the nature of the task suggests that participants must follow the cue information (therefore must not neglect the cue related information) to make a correct response whilst repeat trials can be correct even when the participant neglects the cue related instruction (goal neglect). What was novel about the letter-monitoring task presented by Duncan and colleagues was that initially the cue took the form of a written instruction of the form “WATCH LEFT” or “WATCH RIGHT”. Following a certain number of letter / digit pairs, the cue took the form of a plus (“ + ”) or minus (“- “) sign, meaning watch left or right, respectively. The latter cue forms were presented only in the later part of a sequence. Duncan and colleagues have shown that those with goal neglect would often not respond to the latter, symbolic cue, ignoring entirely the instructed switch; and this despite being able to report the meaning of the cue both prior and subsequent to task completion.

Duncan et al. have linked the new attentional capacity to the frontal lobes by showing that goal neglect was pronounced in frontal lobe patients. Whilst Duncan et al. did not describe where in the frontal lobes the patients’ lesions were, their later contention that goal neglect is a component of working memory capacity for maintaining task-related information (e.g. Braver and Cohen 2001; MacDonald et al. 2000) predicts a role for the left DLPFC.

Models of working memory suggest that the dorsolateral prefrontal cortex (DLPFC) is involved in monitoring and manipulating cognitive representations (Duncan and Owen 2000; Koechlin et al. 2003; Owen et al. 1996; Petrides 2000, 2005; Petrides et al. 2012). fMRI studies have revealed increased DLPFC activity during various working memory processes such as: (1) when the information to be maintained constrains short-term memory capacity; (2) during delay intervals when no information is provided (Courtney et al. 1997; Zarahn et al. 1999); (3) when the manipulation of maintained information is required (D’Esposito and Postle 1999; Postle et al. 1999; Rypma and D’Esposito 1999); (4) when participants need to maintain information during a delay period (D’Esposito et al. 2000; Postle et al. 1999; Rypma and D’Esposito 1999) and (5) before selecting an appropriate response following stimulus presentation and task-set maintenance (posterior DLPFC: Burgess et al. 2010). Consistently, fMRI studies also reported DLPFC activation during the goal maintenance phase of various cognitive tasks (Braver and Cohen 2001; Lopez-Garcia et al. 2016; MacDonald et al. 2000; MacDonald III and Carter 2003; Paxton et al. 2008).

Hence, it is reasonable to argue that DLPFC is involved in goal neglect since it represents a failure to maintain task goals in working memory. Despite the studies suggesting the role of DLPFC on maintenance of task-related information (e.g. Braver & Cohen 2001; Unsworth et al. 2012; Spillers et al. 2012; Friedman and Robbins 2022), to the best of our knowledge, the literature is very limited in terms of supporting the link between working memory (and thus DLPFC) and goal neglect, except for a recent study by Yanaoka et al. (2020) who reported a link between goal neglect and right prefrontal regions in pre-schoolers using fNIRS. Following Duncan et al. (2008) proposal, if goal neglect is an example of a failure of working memory for goal maintenance, there should be an observed difference in goal neglect following modulation of DLPFC functioning. Such alterations in performance can be achieved using brain stimulation techniques such as transcranial magnetic stimulation.

Transcranial magnetic stimulation (TMS) offers a non-invasive technique for direct modulation of the human brain (Luber and Lisanby 2014) via the modulation of the neurons in the targeted brain area. Repetitive TMS (rTMS) refers to the long-term stimulation via trains of stimulation with intervals between the trains (inter-train interval: ITI). The intensity of rTMS is known to affect the direction of cortical excitability. The lower frequencies (< 1 Hz) are known to disrupt cortical functioning whilst higher frequencies (> 1 Hz) lead to an enhancement (e.g. motor cortex stimulation: Pascual-Leone and Hallett 1994). The effect of rTMS has been shown to last post-stimulation for several minutes and up to 1 h (Brunoni and Vanderhasselt 2014; Fregni and Pascual-Leone 2007; Maeda et al. 2000; Peinemann et al. 2004). Although initially used for therapeutic purposes in psychiatry and neurology (Hoy and Fitzgerald 2010; McKinley et al. 2012), using TMS on healthy populations is a promising avenue for exploring underlying brain function permitting as it does the discovery of causal links between brain and behaviour (Luber and Lisanby 2014).

TMS stimulation of the brains of healthy individuals has been shown to successfully improve cognitive functioning (visual spatial attention: Hilgetag et al. 2001; Thut et al. 2004; visual search: Hodsoll et al. 2008; mental rotation: Klimesch et al. 2003; analogical reasoning: Boroojerdi et al. 2001; phonological recall: Kirschen et al. 2006; drawing abilities: Snyder et al. 2003; Young et al. 2004; mathematics, calendar calculating and proofreading Young et al. 2004). In particular, high-frequency rTMS to DLPFC has been shown to improve performance on a variety of cognitive tasks (Hwang al. 2010; Vanderhasselt et al. 2006, 2009; Parris et al. 2021). For example, Vanderhasselt, De Raedt, Baeken, Leyman, and D’haenen (2006) reported decreased reaction times on the Stroop task following high-frequency rTMS stimulation at 10 Hz compared to a sham condition. In their sham condition, the same parameters were set but the stimulation region of the TMS coil did not touch the scalp; instead the figure of 8 coil was rested on the scalp on its edge. Hence, the actual stimulation does not occur, but participants’ experience in the two conditions is very similar. TMS has frequently been used for cognitive enhancement (Andrews et al. 2011; Dresler et al. 2013; Fregni et al. 2005), and high-frequency rTMS has been shown to be a promising technique for working memory enhancement (Brunoni and Vanderhasselt 2014; Esslinger et al. 2014; Gaudeau-Bosma et al. 2013; Guse et al. 2013; Koch et al. 2005).

Importantly, researchers have reported increased performance on working memory tasks following high-frequency rTMS procedures. Using 10 Hz rTMS to left and right DLPFC, Bagherzadeh et al. (2016) reported improved performance on verbal working memory tasks. Combined with fMRI, Esslinger et al. (2014) used 5 Hz rTMS to the right DLPFC during the 2-back task. They found faster responses following stimulation compared to the sham condition. Whilst the stimulation did not modify DLPFC activation itself, significantly increased connectivity within the working memory network during the N-back task was reported. Preston et al. (2010) further conducted 10 Hz rTMS to left and right DLPFC and found increased RT performance in the Sternberg paradigm following stimulation compared to the pre-stimulation baseline. Further studies also showed disrupted performance following low frequency rTMS to bilateral DLPFC during 2-back task (Mottaghy et al. 2002) and delayed recognition task (Postle et al. 2006). Thus, if goal neglect is a function of DLPFC working memory capacity, DLPFC stimulation should modify it. This would be particularly interesting given the theoretical involvement in working memory in producing goal neglect (Duncan et al. 2008). If the present results show an alteration in working memory performance (N3-back) together with the changes in the letter-monitoring task performance, it would provide strong evidence for the link between DLPFC, working memory and goal neglect.

In summary, research has revealed a role for DLPFC in working memory performance (e.g. Burgess et al. 2010), especially those reporting increased performance in working memory tasks following high-frequency TMS stimulation over DLPFC (e.g. Bagherzadeh et al. 2016). However, the literature linking goal neglect and DLPFC-supported working memory is limited. Thus, the aim of the present study was to investigate whether high-frequency TMS can alleviate goal neglect observed during the letter-monitoring task. If as hypothesised, goal neglect is a result of a working memory failure to maintain task-related goals, facilitating DLPFC performance using high-frequency rTMS should result in decreased goal neglect. A direct link between left DLPFC and goal neglect would represent a novel and theoretically important finding. We further argue that, due to the nature of the letter-monitoring task, the need to maintain task goals is more important on switch trials where participants must follow the cue-instructed goal to provide a correct response, in contrast to the repeat trials where participants could make a correct response even when the cue information is neglected. Therefore, we hypothesise that if active stimulation of the DLPFC is specifically related to enhanced goal maintenance and not a non-specific increase in task performance relative to sham stimulation, the TMS-related modulation should be primarily observed for the switch trials. To ensure the part of the DLPFC responsible for working memory was successfully targeted, we also aimed to replicate previous literature showing improved performance on the n-back task following high-frequency TMS (e.g. Bagherzadeh et al. 2016) to the DLPFC. Finally, we employed a measure of fluid intelligence to act as a covariate in our analyses.

## Method

### Participants

Twenty-six participants (fourteen females) aged between 19 and 35 (*M* = 26.15, *SD* = 4.55) participated in this study. Participants provided written informed consent and completed a TMS screening form (Rossi et al. 2009) following the information about the TMS procedure and the experiment. In addition to Rossi et al.’s (2009) recommendations, TMS screening form also included additional questions on the use of psychotropic drugs and alcohol in the last 24 h in addition to having adequate sleep. Participants had to pass all the screening questions to be eligible to take part in the study. Data collection was conducted in accordance with the ethical approval from Bournemouth University Ethics Committee. None of the subjects had any medical conditions or contraindications for rTMS (Rossi et al. 2009; Wassermann 1998).

### Materials

#### Letter-monitoring task

**Fig. 1:**
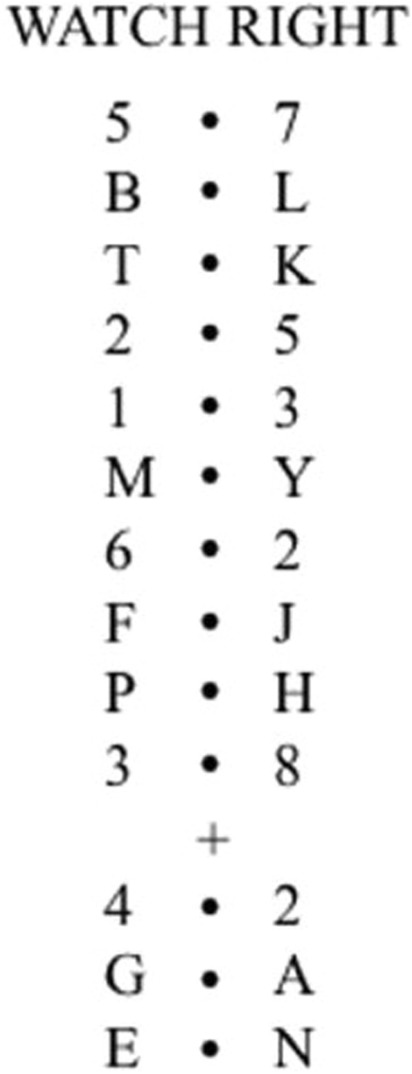
An example demonstration of a letter-monitoring task trial taken from Duncan et al. (1996). Starting from the top to bottom, “Watch RIGHT” message (1 s) is followed by the pairs. Each pair is presented in a separate screen for 200 ms with a blank interval of 200 ms

The letter-monitoring task was taken from Duncan et al. (1996) as a measure of goal neglect. Participants were first seated in front of the screen with a distance of 40 cm and asked to focus on the centre of the screen until the initial message. In the letter-monitoring task, participants are presented with pairs of letters or digits on the left and right side of a central dot. The task is to ignore the digit trials and read aloud the letters on the directed side on the letter trials. The task was designed and administered using Experiment Builder software (SR Research Experiment Builder 2.^3^.^1^ [Computer software], 2020). Stimuli were presented in black against white background using Times New Roman (bold) with 24 pt. using 17-inch screen. Each “trial” included the presentation of 13 individually presented pairs of digits/letters (Fig. 1). Digits were chosen from the set 1–8, and letters were randomly chosen without replacement from the letters of the alphabet (except D, I, O, V, and W). Following the instructions of Duncan et al., participants were first prompted by a “READY?” message. Following the participant’s positive response via verbal report, the experimenter made a key press to initiate a 500 ms blank interval after which the practise trial began. Each trial started with the presentation of the instruction “WATCH LEFT” or “WATCH RIGHT” for 1 s indicating the side from which the participant was required to report the letters. The “WATCH LEFT/ RIGHT” message was followed by a blank screen presented for 1000 ms to allow participants to get ready for the upcoming stimulus sequence. Each stimulus screen consisted of either a pair of digits or letters presented for 200 ms followed by a blank interval of 200 ms. Initially, there were ten pairs. After the 10th pair, the cue with a “ + ” or “- “ symbol was presented in the centre of the screen for 200 ms. A “ + ” sign indicated to the participant that they must attend to the right whilst “-” sign indicated to attend to the left side of the dot (again reporting only from trials with letters). Following a further 200 ms, three more pairs were presented. After the symbol, the first pair was always digits and the last two were always letters. Thus, each trial had total of 13 pairs of digits or letters. Please see Fig. 1 for an example trial. A scoring sheet with correct answers was prepared for the experimenter in advance to manually record the participant’s responses.

To ensure that the meaning of the cue was remembered correctly, pieces of paper were placed on the appropriate side of the computer monitor with “PLUS” (for the right) and “MINUS” (for the left) signs written on them. All participants were instructed to: (1) read aloud the letters and ignore the digits; (2) initially report from the side instructed by the message on the screen (until the cue is presented); (3) then use the cue (+ or—sign) to attend the correct side for the next three pairs. The task comprised 2 blocks of 12 experimental trials (with 13 pairs presented successively on each trial) with 3 sub-blocks (4 trials each) within each block. Participants also received a practise trial which was repeated until at least one letter was reported from either (correct or incorrect) side and the “ ± “ cue was reported accurately. For some trials, participants had to change the attended side (e.g. a WATCHLEFT message followed by the + cue which indicates attending right) whilst others did not require a change (e.g. a WATCHLEFT message followed by the—cue which indicates attending left). To equalise the number of trials with change and no change, in each successive trial of four, there was one “WATCH LEFT” followed by a “-” cue, one “WATCH LEFT” followed by a “ + ” cue, one “WATCH RIGHT” followed by a “-” cue and one “WATCH RIGHT” followed by a “ + ” cue presented in random order. Participants were asked to repeat the rule again between each block. Instructions for the task were provided following Duncan et al.’s (1996) instructions.

*Scoring* A correct response was defined as the following of the cue instruction. Participants received a score of one for each letter reported from the correct side. A perfect trial included a score of two with two letters reported from the correct side. There were two requirements for trials to be counted. First, for trials to be valid, participants had to report at least three letters from the appropriate side indicated by the initial message (“WATCH LEFT”/ “WATCH RIGHT”) from the first five presented pairs. This was to ensure that participants attended to the side indicated by the initial message. Second, participants had to report at least one valid change and one valid no-change trial to pass each sub-block. Participants received a score of 0 for each failed sub-block. The final score was computed by averaging the scores and transforming into percentages. Scores indicate to what extent a participant’s performance was affected by the cue.

#### N3-back task

N3-back task consisted of black letters using the font Mono with 28 px presented on a white background at the centre of a 17-inch screen. Participants were asked to indicate as quickly and as accurately as possible if the current letter presented was the same as the letter presented three trials ago. Participants were asked to press “1” for a “no”, and press “3” for a “yes” response. Stimuli were presented on the screen until a key press or maximum duration of 2500 ms. In between the letters, participants were also presented with a fixation cross using the font Mono with 28 px for 2000 ms. There was a block of 40 pseudo randomly presented letters (chosen randomly from 4 alternative list) with a block of 20 practise trials. The task was designed and presented using OpenSesame software version 3.2.8 (Mathôt et al. 2012). Each participant completed the N3-back task following active and sham stimulations. Accuracy was calculated separately for stimulation and sham sessions.

#### Matrix reasoning

We used the matrix reasoning subtest of Wechsler Adult Intelligence Scale-Fourth Edition (Wechsler, 2008) as a measure of fluid abilities. Participants were asked to perform matrix reasoning subtest following the instructions provided in the manual.

#### The profile of mood states (POMS)

We used abbreviated version of POMS to measure the effect of TMS on the mood (Vanderhasselt et al. 2006). Administration of POMS required participants to rate how they feel ‘‘at this moment’’ using five-point scale (0–4). POMS includes 40 items with the subscales of tension, anger, fatigue, depression, esteem-related affect, vigour and confusion. A total mood disturbance is calculated by subtracting the totals for the positive subscales from the sum of negative subscales.

### TMS parameters

The stimulation procedure was carried out using a MAGSTIM high-speed stimulator (Magstim Company Limited, Wales, UK) with a figure-8-shaped coil. High-frequency rTMS procedure was used to stimulate left DLPFC. We used 10–20 electrode positioning system to locate F3 for left DLPFC (Herwig et al. 2003). The coil was placed at 45° from the midline (orientation). Before each condition (active and sham), resting motor threshold was determined for each participant following an observation of 50 µV peak-to-peak amplitude in at least five out of ten attempts measured via the electrodes over the right abductor pollicis brevis muscle (Tranulis et al. 2006). Stimulus intensity was 110% of rMT using 10 Hz stimulation frequency. There were total number of 40 4-s trains with 26 s inter-train-interval. The stimulation procedure lasted 20 min with total number of 1600 stimulations. The sham condition involved programming the same parameters, but the coil was held at an angle of 90 degrees, only resting on the scalp with one edge (as per Vanderhasselt et al. 2006).

### Design and procedure

Data collection took place in a designated quiet room with air conditioning facilities in one of Bournemouth University’s Psychology Research Booths. Participants were provided with an information sheet and a short demonstration of the TMS procedure. In their first session, and following informed consent, participants were asked to perform matrix reasoning task before the stimulation/sham procedure. Participants then completed the TMS safety screening questionnaire (Rossi, 2009), followed by a block of the letter-monitoring task to make sure they were familiar with the task. Participants then underwent 20 min of stimulation/sham stimulation. Following this, we asked each participant to complete three blocks of the letter-monitoring task. Two different versions of the letter-monitoring task were used for each participant in the stimulation and sham stimulation conditions. Participants were told that some stimulation procedures may create a sensation whilst others may be unnoticeable, and that they would receive two different types of stimulation in two separate sessions. Versions were the same except for the randomised order of pairs of digits/letters. After completion of the letter-monitoring task, participants were asked to complete a block of N3-back task. The order of conditions (active vs sham) and task versions were counterbalanced across participants and was single blind. Therefore, a single blind, within-subjects design was used. The letter-monitoring task and the matrix reasoning test took around 10 min each whilst N3-back task took a maximum of 5 min to complete. Participants were also asked to complete the POMS to measure their mood at the end of each session.

**Fig. 2:**
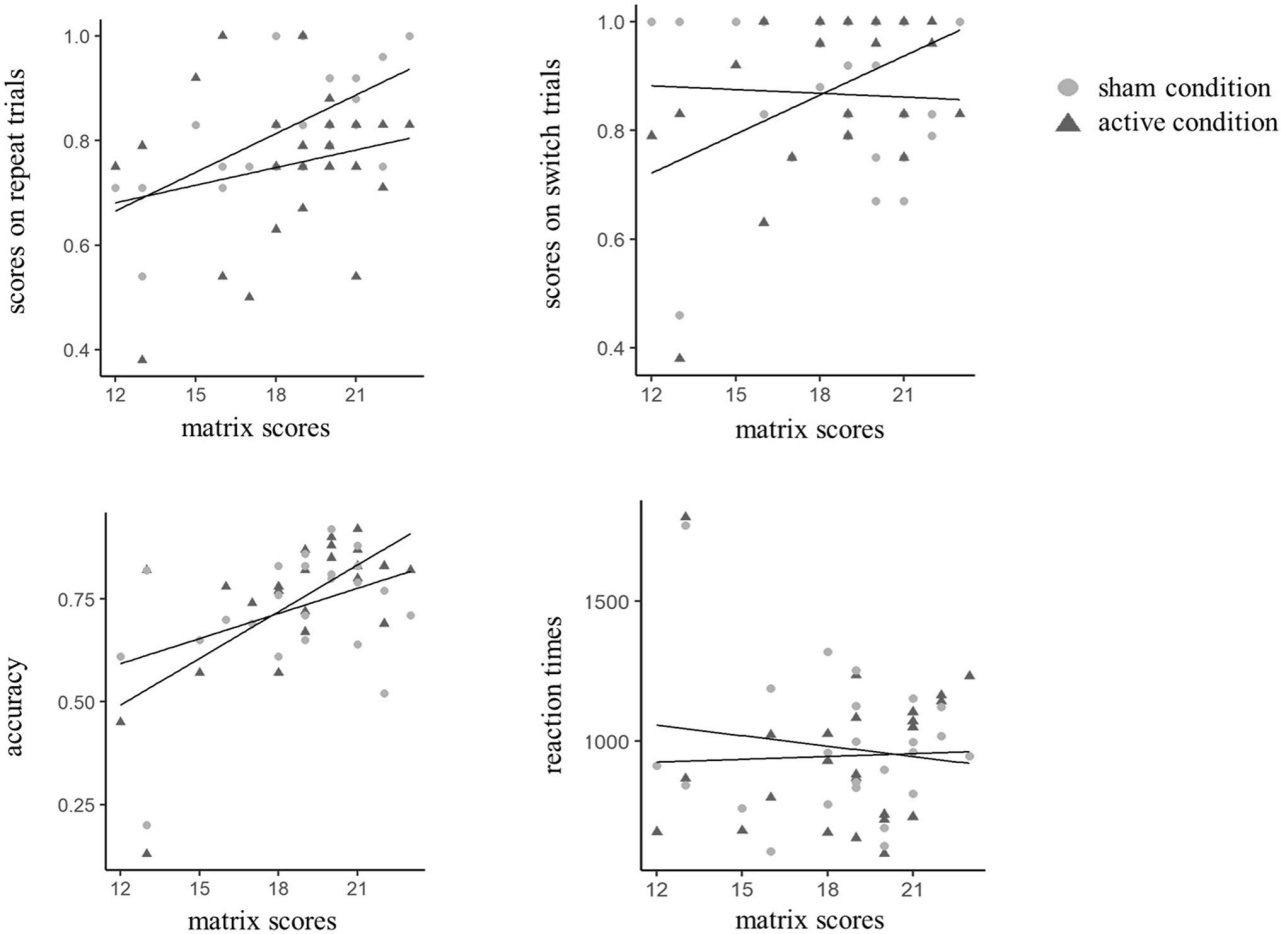
Performance on letter-monitoring task (top) and N3-back task (bottom) by condition (active and sham)

## Results

To investigate the results from the letter-monitoring task, a 2 (stimulation: active and sham) X 2 (condition: repeat and switch trials) repeated measures ANCOVA (with the matrix scores and age as the covariants) revealed that the stimulation main effect was not significant (*F*(1, 23) = 1.22, *p* = 0.281) with no interaction with the matrix scores (*p* = 0.409) and age (*p* = 0.732). The Condition main effect was also not significant (*F*(1, 23) = 0.82, *p* = 0.373) with no interaction with the matrix scores (*p* = 0.099) and age (*p* = 0.110), indicating that there was no statistically significant difference between the repeat and switch trials when controlling for age and matrix scores. However, the Stimulation X Condition interaction was significant (*F*(1, 23) = 7.95, *p* = 0.01, *η*^2^ = 0.26). When the covariates were included, the analysis revealed no additional interaction with age (*p* = 0.397) but a further interaction with matrix reasoning (*F*(1, 24) = 9.59, *p* = 0.005, *η*^2^ = 0.29).

**Fig. 3:**
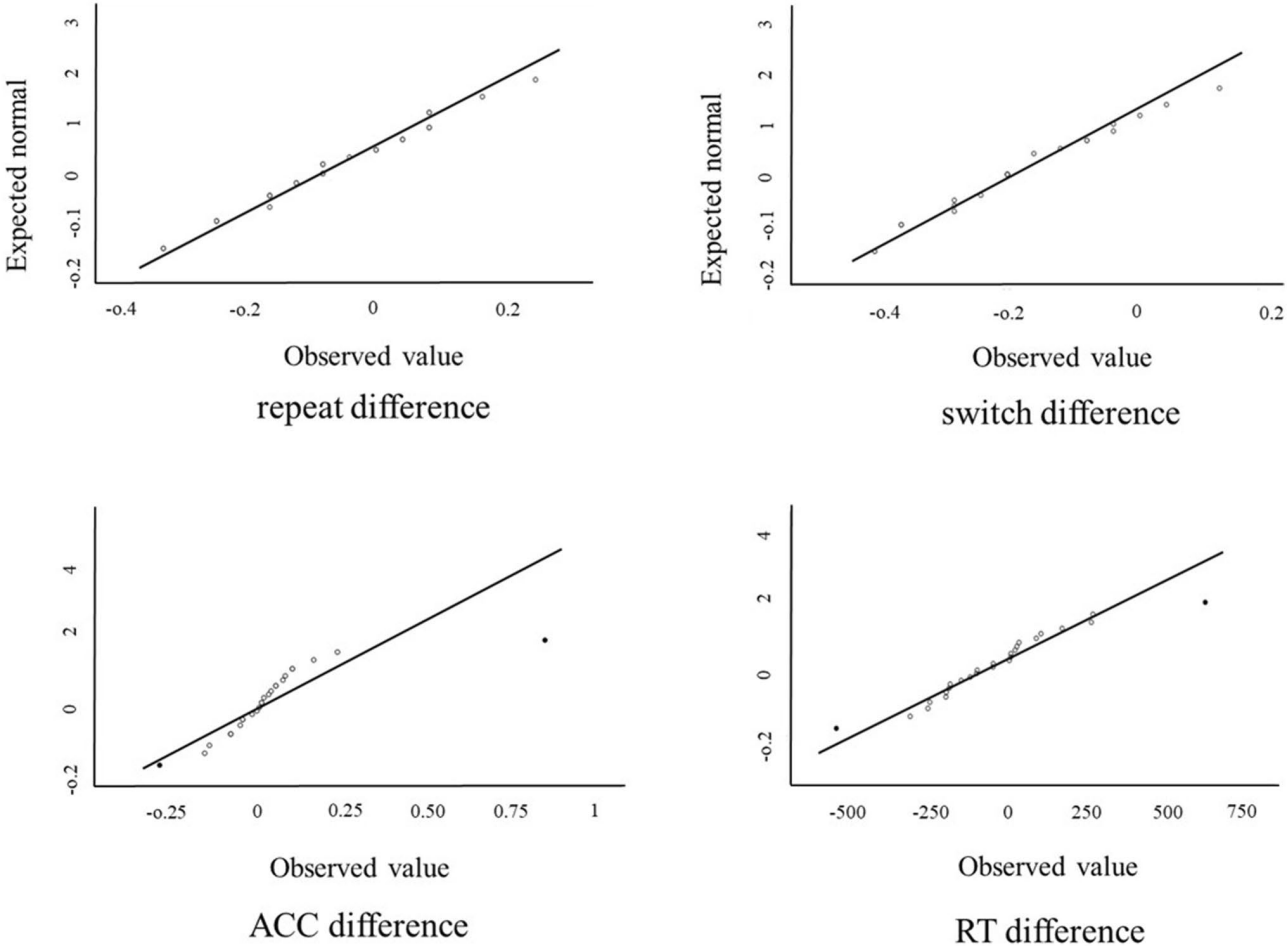
Q–Q plots for the score difference between active and sham stimulation for the letter-monitoring task (top) and N3-back task (bottom). Outliers are indicated in black

To explore the Stimulation X Condition interaction, we compared the scores between active and sham stimulation conditions whilst also adjusting for age and the matrix scores. Results of the ANCOVA revealed that, following stimulation, the scores on the switch trials increased with the matrix scores (as covariate) compared to the sham condition, (*F*(1, 23) = 8.68, *p* = 0.007, *η*^2^ = 0.27; see Fig. 2), whilst the effect of age was not significant (*p* = 0.368). There was no significant difference between active and sham conditions for the repeat trials [*F*(1, 23) = 0.71, *p* = 0.408], and there was no effect of age (*p* = 0.804) and matrix scores (*p* = 0.247) (see Fig. 3 for the effect on stimulation for the letter monitoring and N3-back task (observed by the difference in the scores following active vs sham condition (score in active stimulation—sham stimulation). Please see Table 1 for the demographic information and the performance for each participant based on stimulation conditions.

**Table 1:** Demographics and scores on each task with mean and standard deviations during active and sham sessions

Letter-monitoring task scores								N-back task				Time of the day	
Active session						Sham session		Active Session		Sham Session		Active	Sham
Participant	Gender	Age	Matrix	Repeat trials	Switch trials	Repeat trials	Switch trials	ACC	RT	ACC	RT		
1	Male	19	16	0.54	0.63	0.71	0.83	0.4	1022.56	0.69	1188.73	Afternoon	Afternoon
2	Male	30	12	0.75	0.79	0.71	1	0.45	675.52	0.61	912.97	Morning	Morning
3	Female	20	13	0.38	0.38	0.54	0.46	0.13	1800.32	0.2	1772.27	Afternoon	Afternoon
4	Male	23	20	0.83	1	0.92	1	0.9	738.24	0.8	690.7	Afternoon	Afternoon
5	Male	26	20	0.75	1	0.83	0.75	0.85	1746.55	0	1096.14	Afternoon	Afternoon
6	Male	21	19	0.75	0.79	0.75	0.79	0.87	880.43	0.86	853.08	Afternoon	Afternoon
7	Male	27	21	0.54	0.75	0.88	0.75	0.83	1071.06	0.79	1153.19	Morning	Evening
8	Male	23	18	0.83	1	1	1	0.78	673	0.83	774.86	Afternoon	Afternoon
9	Female	25	20	0.88	0.96	0.79	0.92	0.88	597.92	0.92	626.35	Afternoon	Afternoon
10	Male	33	18	0.63	0.96	0.75	0.96	0.77	1026.03	0.76	1320.32	Afternoon	Afternoon
11	Female	23	16	1	1	0.75	1	0.78	798.65	0.7	606.46	Morning	Morning
12	Male	29	19	1	0.83	0.83	0.92	0.72	1083.9	0.86	1253.14	Afternoon	Afternoon
13	Female	25	19	0.67	0.83	1	1	0.82	653.68	0.83	833.54	Afternoon	Afternoon
14	Male	28	13	0.79	0.83	0.71	1	0.82	866.61	0.82	843.43	Afternoon	Afternoon
15	Female	25	19	0.79	0.79	0.75	0.83	0.72	867.85	0.71	998.43	Afternoon	Afternoon
16	Female	34	21	0.83	1	0.92	1	0.92	1050.36	0.83	996.32	Morning	Morning
17	Male	23	22	0.83	1	0.75	0.79	0.83	1142.59	0.77	1018	Afternoon	Afternoon
18	Female	35	19	0.75	1	1	0.92	0.67	1235.4	0.65	1125.95	Afternoon	Afternoon
19	Female	25	23	0.83	0.83	1	1	0.82	1232.45	0.71	946.84	Afternoon	Afternoon
20	Female	24	15	0.92	0.92	0.83	1	0.57	680.04	0.65	759.59	Afternoon	Evening
21	Male	25	17	0.5	0.75	0.75	0.75	0.74	589.68	0.69	1120.35	Afternoon	Afternoon
22	Female	31	21	0.75	1	0.88	0.83	0.87	1103.89	0.64	812.89	Morning	Morning
23	Female	22	21	0.83	0.83	0.83	0.67	0.8	729.19	0.88	960.38	Afternoon	Afternoon
24	Female	27	22	0.71	0.96	0.96	0.83	0.69	1163.83	0.52	1122.14	Morning	Afternoon
25	Female	35	20	0.79	1	0.83	0.67	0.85	719.54	0.81	898.3	Afternoon	Evening
26	Female	22	18	0.75	1	0.83	0.88	0.57	930.4	0.61	959.14	Evening	Evening
Mean		26.15	18.54	0.76	0.88	0.83	0.87	0.73	964.60	0.70	986.29		
SD		4.55	2.90	0.145	0.15	0.11	0.14	0.18	311.67	0.20	245.24		

We then investigated the scores for the N3-back task whilst controlling for the matrix scores due to the well-established link between working memory and fluid abilities (Unsworth et al. 2014). Four participants’ scores were removed as outliers following Q-Q plots (see Fig. 3). The data were still counterbalanced after the removal of the outliers.

Analysis of covariance revealed that following DLPFC stimulation, the accuracy on the N3-back task increased with the matrix scores (*M* = 0.74, *SD* = *0.1*7) compared to the sham condition (*M* = 0.73, *SD* = *0.1*5)(*F*(1, 21) = 9.24, *p* = 0.006, *η*^2^ = 0.31), whilst the effect of age was not a significant (*p* = 0.47). Analysis of covariance with the reaction time data during N3-back when matrix scores and age were covariates was not significant (*F*(1, 21) = 0.97, *p* = 0.335).

Pairwise comparisons also revealed that there were no significant mood differences between active (*M* = 15.58, *SD* = 13.63) and sham (*M* = 14.81, *SD* = 12.24) stimulation conditions, *t(*25) = 0.47, *p* = 0.644).

## Discussion

This study investigated the effects of high-frequency rTMS of left DLPFC on goal neglect with the aim of investigating whether the attentional limit that produces goal neglect is related to DLPFC-supported working memory. We used 10 Hz stimulation over left DLPFC for 20 min. In a different session, the same participants also underwent a sham stimulation where the exact parameters were employed but the TMS coil did not touch to the scalp in a way that would enable active stimulation. To ensure we modulated DLPFC-supported working memory capacity, participants were asked to complete an N3-back task as well as the task we employed to measure goal neglect: the letter-monitoring task (Duncan et al. 1996).

We found that following stimulation of the left DLPFC, experiences of goal neglect (indicated by higher scores for the switch trials on the letter-monitoring task) were reduced with the influence of fluid intelligence compared to the sham condition. We further found that the accuracy on N3-back performance was also increased with the influence of fluid intelligence. This provides support for a link between the DLPFC and this new attentional capacity: the ability to construct, maintain, and appropriately weight all components of goal representations over an extended period and hence not to neglect the goal. Importantly, reduced goal neglect with the fluid intelligence was only observed on switch trials whilst there was no significant difference between active and sham conditions on repeat trials. That is, following DLPFC stimulation, the accuracy increased with fluid intelligence on the critical switch trials on which following the cue related goal was needed for a correct response. Such a pattern of results indicates the effect of stimulation was specific to trials on which goals could be neglected and was not a general effect of TMS across all the tasks. Such a finding is consistent with the literature suggesting a role for DLPFC in maintaining goal-related information (Braver and Cohen 2001; Lopez-Garcia et al. 2016; MacDonald et al. 2000; MacDonald III and Carter 2003; Paxton et al. 2008) with the additional data stressing the effect of fluid intelligence for benefitting the stimulation.

This finding is also consistent with Duncan et al. (2008) suggesting that working memory is needed to maintain the goal-related information in the task model. Duncan et al. suggested that, unlike traditional working memory measures, which focus on keeping information readily accessible whilst performing a secondary task, the letter-monitoring task requires keeping the goals available throughout the task and the ability to handle competition between different aspects of the task model, and therefore represents a new kind of attentional limit. They suggested that to be able to perform the task, instructions must be turned into task goals where stimulus–response associations are established. When these goals, or components of them, are lost from the task model, participants fail to follow the goal despite being able to describe it. This type of behaviour was originally reported on frontal lobe damage patients (Luria 1966; Milner 1963). The current study demonstrates that the occurrence of goal neglect can be alleviated following high-frequency DLPFC stimulation, hence providing a corroborating direct link between left DLPFC and goal neglect. This is also consistent with the previous findings of an fNIRS study linking GN to prefrontal regions amongst pre-schoolers (Yanaoka et al. 2020).

**Fig. 4:**
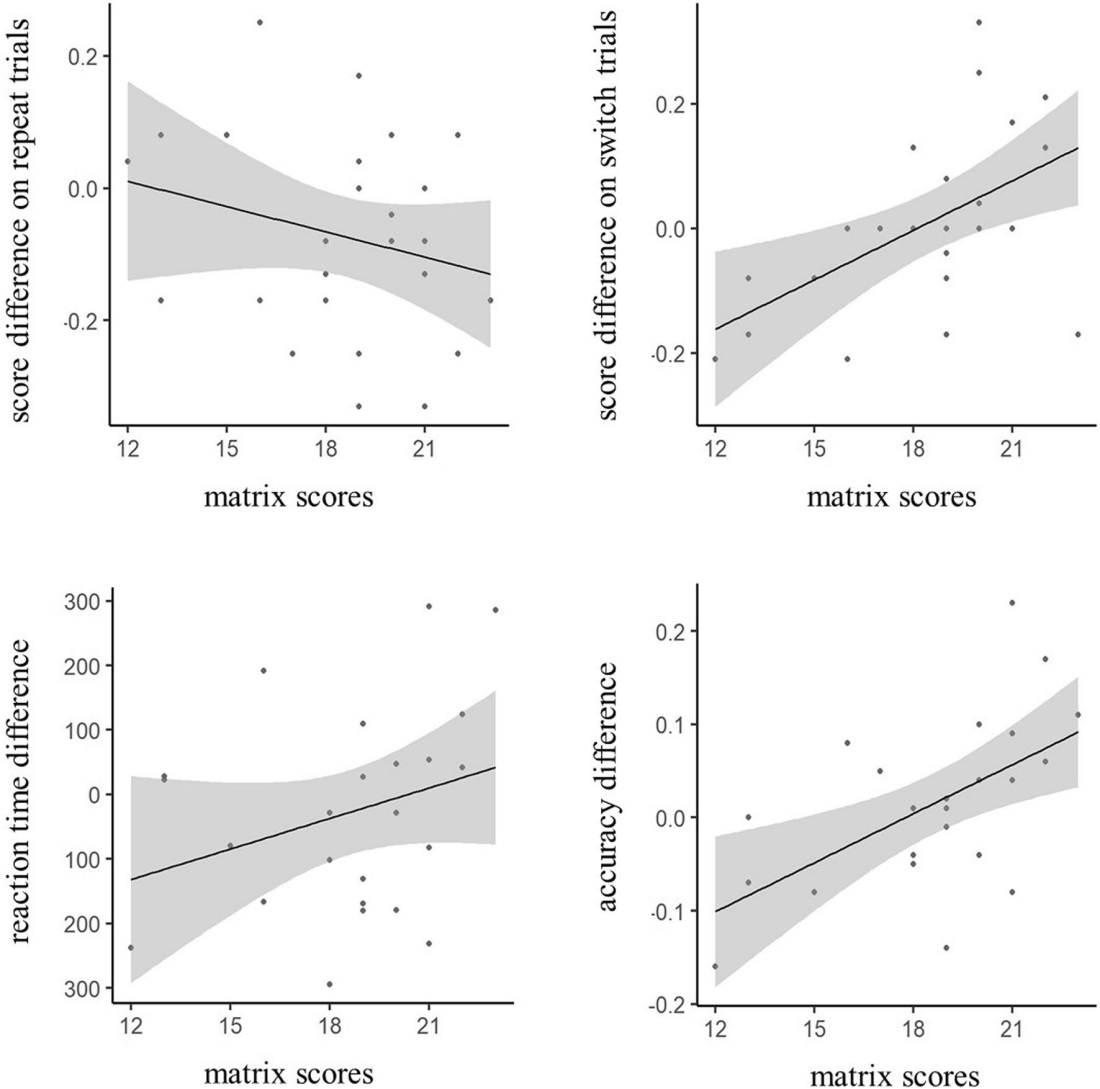
Scatter plot showing the difference in the performance between active and sham conditions (active-sham) for letter-monitoring task (top) and N3-back task (bottom) against matrix reasoning performance

Another important finding from the present study was that the benefit of active stimulation on switch performance was observed mainly in those with high fluid intelligence abilities—as assessed by a matrix reasoning measure. This was not seen in the analysis of repeat trial performance. Thus, in the present study, high-frequency TMS alleviated goal neglect only in those with higher cognitive abilities. This finding indicates that the alleviation of goal neglect following TMS stimulation might be dependent on having the extra, but unused capacity to benefit. Goal neglect has been found to be more likely in those with lower fluid intelligence abilities (Duncan et al. 1996), but our results suggest it is possible for those with higher fluid abilities to experience goal neglect and that their goal neglect can be mitigated, Fig. 4. A goal of future research would be to confirm this finding and to identify where the cut-off is for those that would benefit from interventions such as TMS.

In the present study we also had participants perform an N3-back task to provide corroborating evidence that stimulation modified working memory performance. Stimulating the same site led to increased accuracy in the N3-back task compared to sham condition which is consistent with the literature reporting alterations in working memory capacity following stimulation (e.g. Esslinger et al. 2014; Bagherzadeh et al. 2016; Preston et al. 2010). It was also interesting to see that the difference between the active and the sham condition for the N3-back accuracy was obtained when controlling for the matrix scores. This is consistent with the well-established link between working memory performance and the fluid abilities (e.g. Unsworth et al. 2014). However, the RTs from the N3-back task did not differ following the stimulation. This is somewhat inconsistent with the findings of (Esslinger et al. 2014) which showed faster RTs following DLPFC stimulation compared to sham condition whilst the accuracy did not differ. However, (Esslinger et al. 2014) stimulation procedure differs from the current study with 5 Hz rTMS stimulation of DLPFC. Thus, it is possible that the difference in the pattern of results could be due to the difference in the frequency of DLPFC stimulation. This is consistent with the findings of Bagherzadeh et al. (2016) showing improved accuracy for N2-back task following 10 Hz stimulation over DLPFC. Consistent to our pattern of findings, Jaeggi et al. (2010) also investigated the RT and accuracy differences in the N-back task performance and revealed that higher accuracy was associated with higher fluid abilities whilst reaction times’ performance was not significant.

An important point of discussion, when evaluating the increased accuracy during the N3-back task and the letter-monitoring task following active stimulation of the DLPFC (where both improvements are related to increased matrix scores), is the possible influence of increased attention. There is a common consensus suggesting the close link between working memory and attention (see Oberauer 2019 for a discussion) where working memory is responsible for directing attentional resources (Duncan et al. 2008). Although DLPFC has traditionally been linked to executive control functions such as working memory (e.g. Burgess et al. 2010) as opposed to the brain regions included in the dorsal attention network, the role of DLPFC stimulation has been shown in various attention studies ( Miler et al. 2018; Lema et al. 2021; Johnson et al. 2007). Therefore, it is possible to attribute increased performance in our and previous studies to increased attention. Indeed, whilst we have followed the lead of previous studies and linked improved N3-back performance to improved working memory, our interpretation of the benefit is also compatible with an account based on attention, given the close link between the two cognitive processes. Indeed, the cognitive phenomena of interest in the present study, goal neglect, has been argued to represent a new form of attentional limit (Duncan et al. 2008), but one that is linked to a component of working memory akin to the episodic buffer in Baddeley’s (2000) working memory model. Thus, this specific form of attention can be thought of as a resource managed by a component of working memory in which different task components compete for attention.

Following previous research (e.g. Bagherzadeh et al. 2016; Bridges et al. 2018; Vanderhasselt et al. 2009), in our sham condition, participants received the same parameters at the same neural location (F3), but the coil was positioned in a way that participants did not receive the actual stimulation. Therefore, although the same stimulation noise was produced, the sensation of the stimulation was absent. Participants did not relay suspicions about the sham vs. stimulation conditions. Whilst we acknowledge that, to participants, the stimulation effect would be different, participants reported being oblivious to the TMS/sham procedures as well as the actual potential effect of stimulation, which could have been either harmful or beneficial to performance. Our participants were naïve to the TMS procedures and the initial information about the brain stimulation did not specify that TMS would result in an increase/decrease in the task performance. Participants were also told that they may or may not experience a stimulation sensation; however, even if there is a sensation, this should never be uncomfortable or painful. They were also told that the stimulation may or may not be felt based on the individual differences, targeted brain area and the stimulation protocol. In addition, if the reported effect was based on expectancy, it would have been observed on the repeat trials and the RT data on the N3-back task as well as the switch trials. Regardless, the use of sham stimulation could still be seen as a limitation of the study and results should be evaluated accordingly.

A limitation to our study was that participants were not screened for the caffeine intake and the menstrual cycle. Although it is possible to argue that such impact on the performance would also be seen in other task-related performance (e.g. repeat trials and the RT data from the N3-Back Task) where no effect has been reported, this could be seen as another limitation of the study. The present study also used 10–20 electrode positioning system to locate F3 for left DLPFC (Herwig et al. 2003). Research suggests that although the use of 1020 system is applicable to TMS studies, the use of neuronavigation based on the MRI images of each participant is recommended for more accurate targeting of brain regions (Herwig et al. 2003).

In sum, the present study has shown that goal neglect was mitigated following high-frequency rTMS of the left DLPFC, mainly in those with high fluid intelligence abilities, suggesting a role for DLPFC in goal neglect. The effect was only observed on switch trials indicating a specific and not a general performance improvement. Furthermore, the same stimulation improved the N3-back task where the improvement was also related to fluid abilities, providing evidence that left DLPFC stimulation resulted in improved working memory. Such a finding supports Duncan et al.’s assertion that goal neglect reflects an impairment in a function underpinned by working memory and, more generally, is consistent with the previous research reporting improvements in the performances on cognitive tasks following high-frequency rTMS (Andrews et al. 2011; Bagherzadeh et al. 2016; Dresler et al. 2013; Fregni et al. 2005; Vanderhasselt et al. 2009). The present findings suggest there is potential to alleviate goal neglect in clinical disorders where it might contribute to reported symptoms such as in inattention (Arabaci & Parris 2019; Elisa et al. 2016) and following frontal lobe damage after stroke or atrophy during ageing (Duncan et al. 1996).

## Data Availability

The datasets analysed during the current study are available on request. Please contact corresponding author.
